# Imprinting disorders: a group of congenital disorders with overlapping patterns of molecular changes affecting imprinted loci

**DOI:** 10.1186/s13148-015-0143-8

**Published:** 2015-11-14

**Authors:** Thomas Eggermann, Guiomar Perez de Nanclares, Eamonn R. Maher, I. Karen Temple, Zeynep Tümer, David Monk, Deborah J. G. Mackay, Karen Grønskov, Andrea Riccio, Agnès Linglart, Irène Netchine

**Affiliations:** Department of Human Genetics, RWTH Aachen, Pauwelsstr. 30, Aachen, Germany; Molecular (Epi)Genetics Laboratory, BioAraba National Health Institute, Hospital Universitario Araba, Vitoria-Gasteiz, Spain; Department of Medical Genetics, University of Cambridge and NIHR Cambridge Biomedical Research Centre, Cambridge, UK; Human Genetics and Genomic Medicine, Faculty of Medicine University of Southampton, Southampton, UK; Wessex Clinical Genetics Service, Princess Anne Hospital, Coxford Road, Southampton, UK; Clinical Genetic Clinic, Kennedy Center, Rigshospitalet, Copenhagen University Hospital, Glostrup, Denmark; Imprinting and Cancer Group, Cancer Epigenetic and Biology Program (PEBC), Institut d’Investigació Biomedica de Bellvitge (IDIBELL), Hospital Duran i Reynals, Barcelona, Spain; DiSTABiF, Seconda Università degli Studi di Napoli, Caserta, Italy; Institute of Genetics and Biophysics—ABT, CNR, Napoli, Italy; Endocrinology and diabetology for children and reference center for rare disorders of calcium and phosphorus metabolism, Bicêtre Paris Sud, APHP, Le Kremlin-Bicêtre, France; INSERM U986, INSERM, Le Kremlin-Bicêtre, France; INSERM, UMR_S 938, CDR Saint-Antoine, Paris, F-75012 France; Sorbonne Universites, UPMC Univ Paris 06, UMR_S 938, CDR Saint-Antoine, Paris, France; 3APHP, Pediatric Endocrinology, Armand Trousseau Hospital, Paris, France

**Keywords:** Imprinting disorders, Imprinted genes, Epimutation, Uniparental disomy

## Abstract

Congenital imprinting disorders (IDs) are characterised by molecular changes affecting imprinted chromosomal regions and genes, i.e. genes that are expressed in a parent-of-origin specific manner. Recent years have seen a great expansion in the range of alterations in regulation, dosage or DNA sequence shown to disturb imprinted gene expression, and the correspondingly broad range of resultant clinical syndromes. At the same time, however, it has become clear that this diversity of IDs has common underlying principles, not only in shared molecular mechanisms, but also in interrelated clinical impacts upon growth, development and metabolism. Thus, detailed and systematic analysis of IDs can not only identify unifying principles of molecular epigenetics in health and disease, but also support personalisation of diagnosis and management for individual patients and families.

## Background

Imprinting disorders (IDs) are a group of congenital diseases characterised by overlapping clinical features affecting growth, development and metabolism, and common molecular disturbances, affecting genomically imprinted chromosomal regions and genes. The term genomic imprinting describes the expression of specific genes in a parent-of-origin specific manner - i.e. they are expressed only from the maternal or from the paternal gene copy, but not biparentally. Disturbances of imprinted genes may alter their regulation (“epigenetic mutation") and dosage and rarely their genomic sequences can be altered (“genetic mutation”).

So far, more than 150 human genes have been shown to be imprinted (for review, http://www.geneimprint.com/site/genes-by-species), but it is likely that more remain to be identified. Imprinting marks, like other epigenetic marks, are re-established at each generation by successive removal and re-establishment in the germ cell lineages, and then in early zygotic development. The critical difference between imprinting marks and all others is that they elude postzygotic reprogramming, and therefore are essentially ubiquitous and permanent in somatic tissues - except for the germline lineage that embarks upon the establishment of the subsequent generation (for review, [1]).

**Fig. 1:**
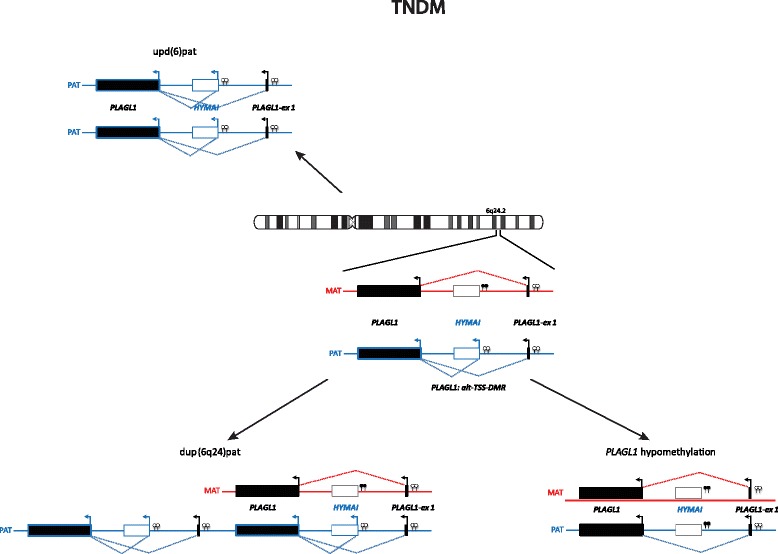
*PLAGL1* imprinted region on chromosome 6q24, altered in TNDM. The currently known imprinted loci associated with one of the known IDs. (Filled boxes, protein coding genes; empty boxes, non-coding genes; Ω miRNAs; filled lollipops, methylated regions; empty lollipops, unmethylated regions; black, genes with biparental expression; red, genes expressed from the maternal (mat) chromosome; blue, genes expressed from the paternal (pat) chromosome; grey, silenced gene copies. Arrows above the genes, transcription direction of sense genes; arrows below the genes, transcription direction of anti-sense genes. IC, imprinting control region)

**Fig. 2:**
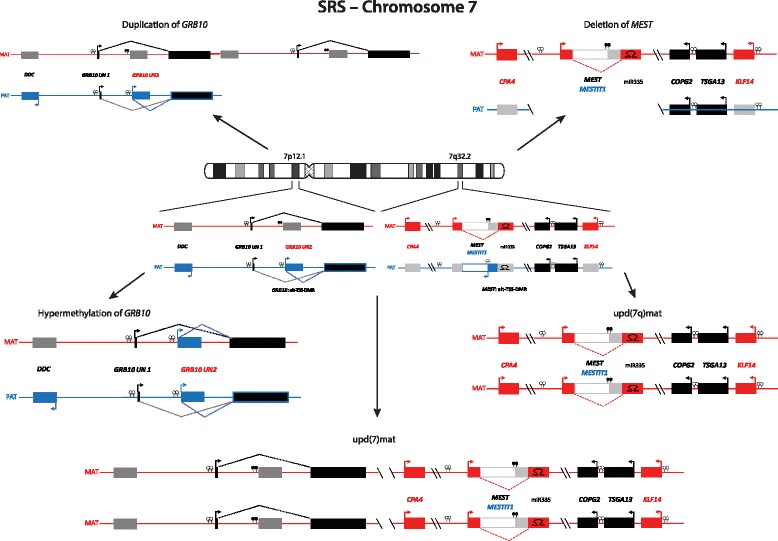
The loci *GRB10* in 7p12.1 and *MEST* in 7q32, affected by (segmental) upd(7)mat or chromosomal imbalances in SRS

**Fig. 3:**
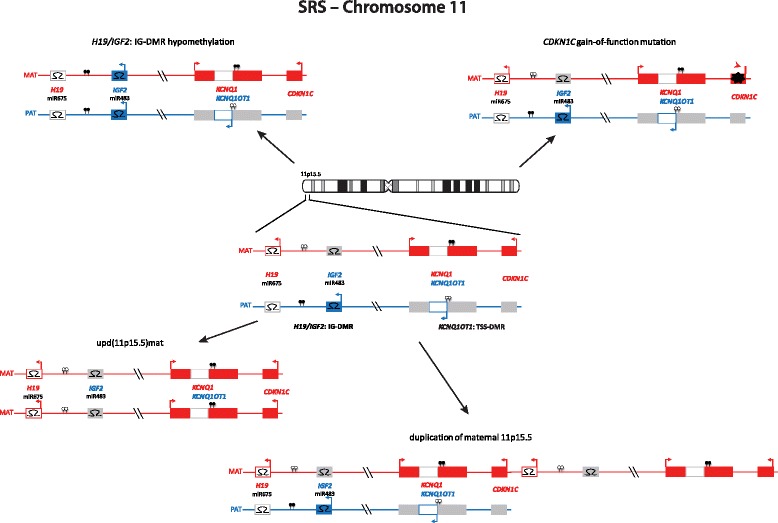
The 11p15.5 cluster can be divided in two functional domains whose imprinting is dependent on distinct imprinting control regions (*H19/IGF2: IG DMR* and *KCNQ1OT1: TSS DMR*). Mainly hypomethylation of the *KCNQ1OT1*: TSS DMR is responsible for SRS

**Fig. 4:**
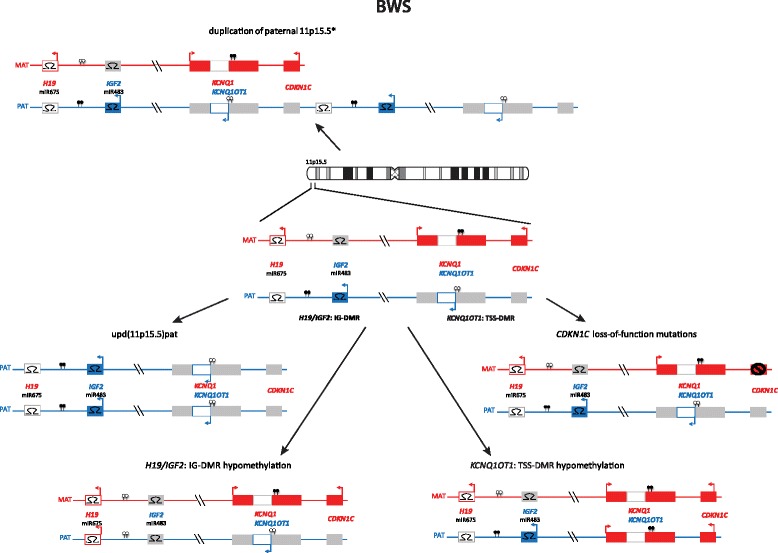
Epimutations and mutations in 11p15.5 are also responsible for BWS

**Fig. 5:**
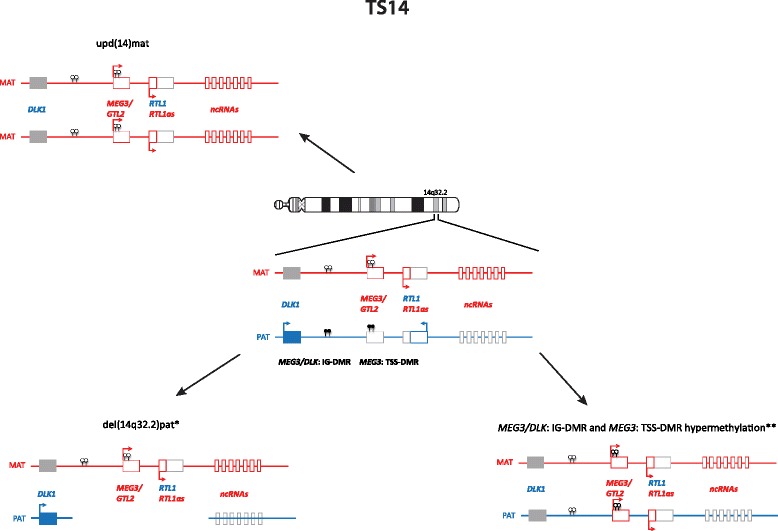
The imprinted region in 14q32.2, and changes associated with TS14

**Fig. 6:**
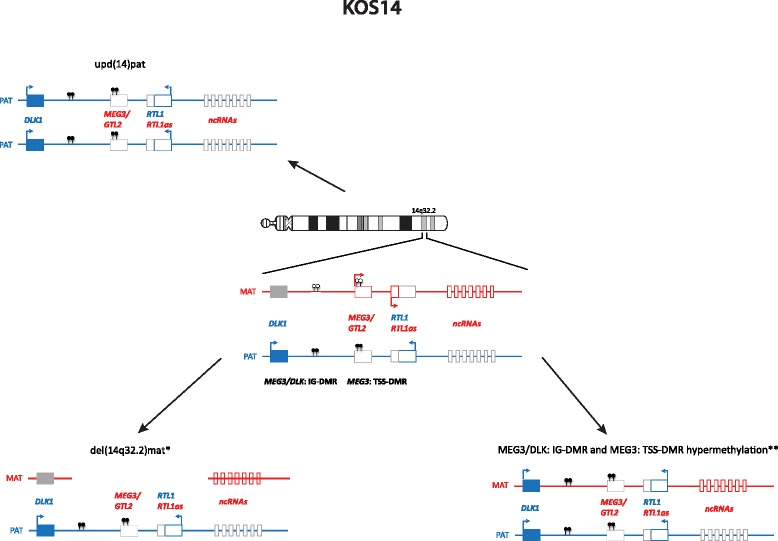
Molecular changes currently known to be associated with KOS14

**Fig. 7:**
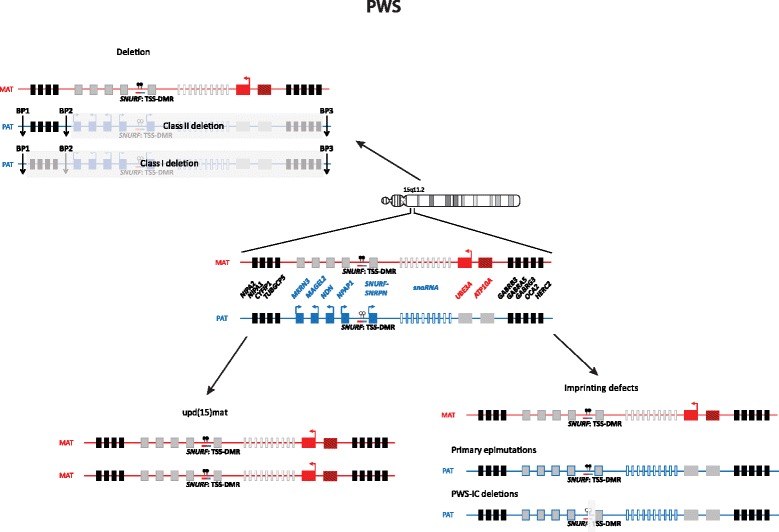
The imprinted region in 15q11.2 and PWS. *UBE3A* encodes an E3 ubiquitin-protein ligase which is expressed exclusively from the maternal allele in human fetal brain and in adult frontal cortex. The role of *ATP10A* is unclear

**Fig. 8:**
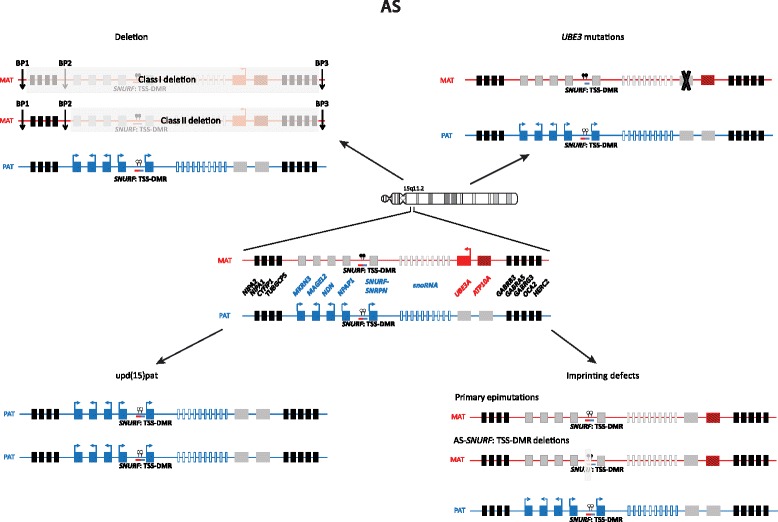
Alterations in 15q11.2 in AS

**Fig. 9:**
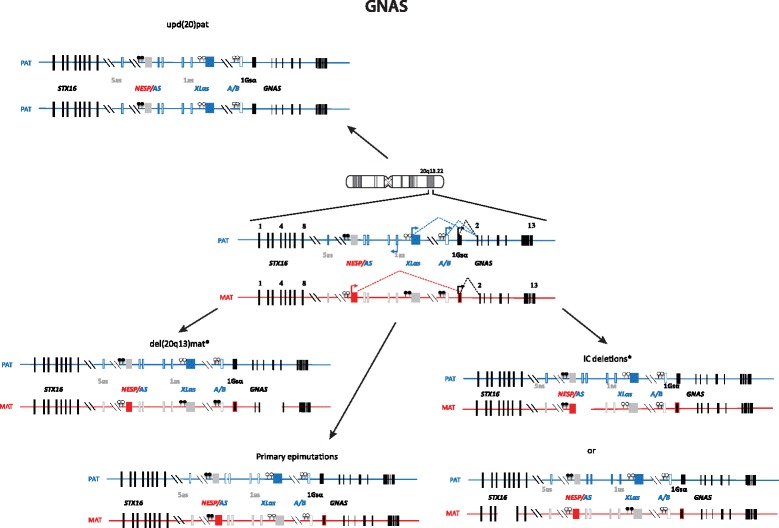
Organization and imprinting of the complex *GNAS* locus at 20q13.22, causing PHP

Imprinted loci often comprise multiple genes under coordinated epigenetic control (Figs. 1, 2, 3, 4, 5, 6, 7, 8 and 9). This control includes four interacting molecular components: DNA methylation, post-translational histone modification, chromatin structure and non-coding RNAs. The intricate interactions of these regulatory mechanisms across development lead to a stage- and tissue-specific transcriptional activity in cells with identical DNA sequences.

In IDs, the regulation of imprinted genes and imprinting clusters are disturbed by different changes. In the majority of ID patients only the disease-specific loci are affected, but an increasing number of individuals have been shown to have disturbed methylation at multiple imprinted loci, the so-called multilocus methylation imprinting disturbances (MLID). Another extreme example of unbalanced imprinting patterns is uniparental diploidy (e.g. complete hydatidiform moles, where all the chromosomes are of paternal origin) or triploidies (e.g. partial hydatidiform moles where an extra haploid set of chromosomes of either maternal or paternal origin is present). These cases are not viable. However, mosaic genomewide uniparental isodiploidy has been reported to be compatible with life (for review, [2]).

## Types of mutations and epimutations in IDs

In the majority of the well established IDs, the same four classes of molecular changes have been reported (Table 1, Figs. 1, 2, 3, 4, 5, 6, 7, 8 and 9): uniparental disomy (UPD), chromosomal imbalances, aberrant methylation (epimutation) and genomic mutations in imprinted genes. The functional result in each case is the unbalanced expression of imprinted genes, but the phenotypic outcome depends on the parental allele affected by the mutation.

**Table 1:** Overview on the molecular findings in the currently known IDs and their clinical characteristics

Imprinting disorder	Prevalence	OMIM	*Chromosomes*/imprinted regions	Type of mutation/epimutation and their frequencies		MLID	Mosaicism	Recurrence risk	Main clinical features
Transient Neonatal Diabetes Mellitus (TNDM)	1/300.000	601410	*6q24*: *PLAGL1*: alt-TSS	upd(6)pat	40 %			<1 %	IUGR, transient diabetes, hyperglycemia without ketoacidosis, macroglossia, omphalocele
				paternal duplications	40 %		No	Up to 50 %	
				methylation defects	20 %	~50 %	Yes	<1 %	
Upd(6)mat	Unknown		*Chromosome 6, 6q16.1qter*	upd(6)mat			Yes	Unknown	
Silver-Russell syndrome (SRS; Russell-Silver Syndrome, RSS)	1/75.000-1/100.000	180860	7	upd(7)mat	~10 %	1 case^a^	No	<1 %	IUGR/PNGR, small prematurely calcified placenta, rel. macrocephaly at birth, hemihypotrophy, prominent forehead, triangular face, feeding difficulties
			*11p15*:	upd(11p15)mat	single case		Unknown	Rare	
				Genome-wide uniparental diploidy	single case		Yes	Rare	
				maternal duplication	<1 %		No	Up to 50 %	
			*H19/IGF2*: IG DMR	hypomethylation	>38 %^a^	7-10 %	Yes	<1 %	
			*CDKN1C*	point mutations	1 family reported		No	In familial cases: up to 50 % in case of maternal transmission	
			*IGF2*	point mutations	1 family reported		No		
Beckwith-Wiedemann syndrome (BWS; Wiedemann-Beckwith syndrome, EMG)	1/15.000	130650	*11p15*:	upd(11p15)pat	~20 %		Yes	<1 %	Pre- and postnatal overgrowth, organomegaly, macroglossia, omphalocele, neonatal hypoglycemia, hemihypertrophy, increased tumour risk
				Genome-wide uniparental diploidy	~2 %		Yes	<1 %	
				chromosomal aberrations	2-4 %		No	Up to 50 %	
			IH19/IGF2: IG DMR*; KCNQ1OT1:* TSS-DMR	hypermethylation	5-10 %		Yes	unclear	
				hypomethylation	40-50 %	25 %	Yes	<1 %	
			*CDKN1C*	point mutations	5 % (sporadic) 40–50 % (families)		No	Up to 50 %	
Kagami-Ogata syndrome (KOS14; upd(14)pat syndrome)	unknown	608149	*14q32:*	upd(14)pat	65 %			in case of RT	IUGR, polyhydramnion, abdominal and thoracal wall defects, bell-shaped thorax, coat-hanger ribs
			*MEG3/DLK1:* IG DMR	maternal deletion	15 %			up to 50 %	
			*MEG3:* TSS DMR	aberrant methylation	20 %			<1 %	
Temple syndrome (TS14; upd(14)mat syndrome)	unknown	616222	*14q32:*	upd(14)mat	78 %			In case of RT	IUGR/PNGR, neonatal hypotonia, feeding difficulties in infancy, truncal obesity, scoliosis, precocious puberty, small feed and hands
			*MEG3/DLK1: IG DMR*	paternal deletion	10 %			Up to 50 %	
			*MEG3: TSS DMR*	aberrant methylation	12 %	1 case^a^		<1 %	
Prader-Willi syndrome (PWS)	1/25.000-1/10.000	176270	*15q11-q13*	paternal deletion	70 %			Up to 50 %	PNGR, mental retardation, neonatal hypotonia, hypogenitalism, hypopigmentation, obesity/hyperphagia
				upd(15)mat	<30 %			In case of RT	
				aberrant methylation	~1 %	1 case		<1 %	
Angelman syndrome (AS)	1/20.000-1/12.000	105830	*15q11-q13:*	maternal deletion	70 %		No	Up to 50 %	mental retardation, microcephaly, no speech, unmotivated laughing, ataxia, seizures, scoliosis
				upd(15)pat	1-3 %			In case of RT	
				aberrant methylation	~4 %		Yes	<1 %	
			*UBE3A*	point mutations	10-15 %		No	In familial cases: up to 50 % in case of maternal transmission	
Precocious puberty (central precocious puberty 2; cppb2)	Unknown	614356	*15q11.2:* *MKRN3*	point mutations	100 %		No	In familial cases: up to 50 % in case of paternal transmission	Early activation of the hypothalamic-pituitary-gonadal axis results in gonadotropin-dependent precocious puberty
Upd(16)mat	Unknown		*Chromosome 16*	upd(16)mat, often associated with chromosomal aberrations			Yes	<1 %	IUGR (40-85 %); heterogeneous, but no specific or unique symptoms
Pseudohypo-parathyroidism (PHP1B, PHP1C, PHP1A)	unknown	603233	*20q13:*	Maternally inherited deletions causing aberrant methylation	8.5 %			Up to 50 % in case of maternal transmission	Resistance to PTH and other hormones; Albright hereditary osteodystrophy, subcutaneous ossifications, feeding behaviour anomalies, abnormal growth patterns
		612462	*GNAS*	isolated epimutations	42.5 %	12.5 %		<1 %	
		103580		upd(20)pat	2.5 %	12.5 %		<1 %	
				maternal and paternal heterozygous loss of function mutations in *GNAS* coding sequence	46.5 %		No	Up to 50 % in case of maternal transmission	
Upd(20)mat syndrome	unknown		*Chromosome 20*	upd(20)mat			No	<1 %	IUGR, PNGR, feeding difficulties

*IUGR* intrauterine growth retardation, *PNGR* postnatal growth retardation

^a^This case carries both upd(7)mat and a TS14 epimutation [82], if studied in different tissues

Since the genetic counseling for each ID is affected by both its familial inheritance and its underlying pathogenetic mechanism, precise molecular diagnosis is essential for accurate management and reproductive counseling. Furthermore, in some IDs somatic and germline mosaicism have been reported, a finding which may be difficult to diagnose, but must be considered since it may compromise molecular genetic testing.

### Uniparental Disomy (UPD)

UPD is the inheritance of both chromosomal homologues from the same parent and has been reported for nearly all IDs (Table 1; for review, [3]). The recurrence risk for another child with UPD is generally low with the exception of those UPDs affecting acrocentric chromosomes (chromosomes 14 and 15): these chromosomes are prone to Robertsonian translocations (RT) which predispose to non-disjunctional errors and thus a UPD formation. However, the risk to have a child with UPD is below 1 %, but prenatal testing for UPD is recommended in carriers of balanced translocations affecting chromosomes carrying imprinted genes [4].

### Chromosomal rearrangements (deletions, duplications, translocations)

Chromosomal imbalances either cause a loss of a gene and thereby a loss of expression of an imprinted gene in case of deletions or translocations or they result in an overexpression by duplication of imprinted chromosomal material. However, due to the complex regulation mechanisms in imprinted regions, loss of chromosomal material can also indirectly cause an overexpression of an imprinted gene due to the removal of a negative cis-acting element and vice versa (e.g. [5–7]).

In some IDs, deletions account for the majority of cases, e.g. in Angelman syndrome (AS) and Prader-Willi syndrome (PWS). They can either occur *de novo*, or they can be caused by inherited chromosomal rearrangements (e.g. RT). In case of familial cases, the parent-of-origin-dependent gene expression results in autosomal-dominant inheritance with a parent-of-origin-dependant penetrance.

### Intragenic mutations in imprinted genes

So far, genomic point mutations in imprinted genes have only been reported for Beckwith-Wiedemann syndrome (BWS), Silver-Russell syndrome (SRS), AS, precocious puberty and pseudohypoparathyoridism (PHP) (Table 1). In precocious puberty syndrome (central precocious puberty 2; cppb2), *MKRN3* mutations are the only causative molecular alterations known so far. In the other IDs, their significance differs: AS mutations in the *UBE3A* contribute to 10–15 % of cases, and approximately 30 % are inherited. In PHP, mutations on the coding maternal allele of *GNAS* are responsible for 70 % of type 1A disease (~50 % of total PHP), whereas, deletions of genomic regulatory regions have been identified in 20–30 % of the 1B subtype (~8.5 % of total PHP) [8]. In BWS, inhibiting *CDKN1C* mutations can be detected, in SRS, only one case with an activating *CDKN1C* mutation has been reported so far [9]. To further determine the recurrence risk in the families of these patients, familial segregation studies should be offered to establish the maternal/paternal inheritance or lack thereof, even when parents do not show obvious clinical features.

### Epimutations

Epimutations aberrant methylation of a differentially methylated region (DMR) without alteration of the same genomic DNA sequence account for up to 50 % of molecular changes in IDs (Table 1). To contribute to the full clinical picture of an ID, hypo- or hypermethylation should affect the disease-specific germ-line DMR (e.g. the *H19*-DMR in 11p15), but in several IDs the methylation at further DMRs (e.g. *IGF2*-DMRs in 11p15) is altered [10] and might influence the severity of an ID (e.g. Kagami-Ogata syndrome/KOS14, [11]). Epimutation can occur as a result of a DNA mutation in a cis- or trans-acting factor (“secondary epimutation”), or as a primary epimutation in the absence of any DNA sequence change (“primary epimutation”). Primary epimutations often occur after fertilization and lead to somatic mosaicism. It has been estimated that the rate of primary epimutations is 1 or 2 orders of magnitude greater than somatic DNA mutations [12] and is associated with assisted reproductive technology [13], in keeping with environmental disturbances.

In terms of molecular mechanism, the four causes of IDs can interact: chromosomal translocations can predispose to both imbalances and UPD, and deletions or point mutations in regulatory domains can affect the imprinting status of a DMR [6, 10, 14]. It is noteworthy that some molecular changes may occur postzygotically, resulting in a mosaic distribution. Mosaicism can obscure genotype-phenotype correlation and is also associated with somatic asymmetry; and discordant monozygotic twinning, which can be regarded as an extreme expression of epigenetic asymmetry, is a common feature in IDs (for review, [15]). It may also render difficult the molecular diagnosis if the analysed tissue is not or poorly epigenetically modified.

## Clinical and molecular findings in Imprinting Disorders

With the exception of the precocious puberty syndrome, the clinical features of IDs are present at birth and in early childhood. Indeed, some of them can be identified prenatally. Each ID is characterised by specific clinical features, and they have been regarded as separate entities. However, the majority of IDs share clinical (and molecular) characteristics (Tables 1 and 2), and in nearly all of them growth, metabolism and/or development are affected. Furthermore, they share several sequelae (e.g. diabetes; Table 2). In several disorders, the symptoms are subtle, unspecific and transient; therefore, some IDs are probably mis- and underdiagnosed.

**Table 2:** Comparison of the major clinical findings in the known and suggested IDs, showing a broad clinical overlap between the different disorders

Congenital ID	TNDM	upd(6)mat	SRS		BWS	TS14	KOS14	PWS	AS	Precocious puberty	upd(16)mat	PHP	upd(20)mat
Reference	[18]	Web^a^	[83]		[33]	[43]	[84]	[52]	[85]	[61]	Web^a^	[86]	[70]
number of patients	155	13	20	44	403	51	34	90			61	63	15
ID specific chromosome	6	6	7	11	11	14	14	15	15	15	16	20	20
clinical overlap with	BWS	SRS	upd(6)mat, TS14, upd(16)mat, upd(20)mat		TNDM, KOS14	SRS, PWS	BWS	TS14	AS infant		SRS, upd(6)mat, upd(20)mat		SRS, upd(6)mat, upd(16)mat
	Major clinical and overlapping findings												
IUGR	Yes	53.8 % (7/13)	70 %	82 %		87 %	1	Rare	No		77 % (47/61)		100 %
prenatal overgrowth					Yes		58.8 % (20/34)		No			Yes	
placenta			Abnormality: 8 %	Abnormality: 35 %	Placentomegaly		Placentomegaly		No				
polyhydramion					Reported		97 % (33/34)		No				
PNGR	Yes	33.3 % (2/6)	65 %	57 %		79 %	36.6 % (11/30)	63 %	No		2 % (1/49)		100 %
overgrowth					Yes		(6.7 % (2/30)		No				
organomegaly					43.8 % (153/349)				No				
Asymmetry			30 %	68 %	33.3 % (126/378	4 %			No				
macroglossia	44 % (54/123)				94 % (379/403)				No			7 % (3/35)	
relative macrocephaly			90 %	70 %		56 %			No				1 case
relative microcephaly		1 case							>80 %				
hypotonia			45 % (*n* = 143) [87]			93 %		88 %	<80 %				1 case
abdominal wall defects	21 % (24/114)	1 case	Rare		62.3 % (250/401)		Omphalocele: 32.3 % (11(34)		No		1 case		
					Exomphalos: 56.8 % (142/250)		diastasis recti: 67.6 % (23/34)						
glycemic disorder	TNDM: 100 %		Hypoglycemia: 24 %	Hypoglycemia: 19 %; diabetes type 2 reported in later life	Hypoglycemia: 43.4 % (162/373)	Hypoglycemia diabetes type 2 reported in later life		Diabetes type 2: 25 %	no				
precocious puberty			Frequent	Frequent	Reported	86 %		4 % [88]	No	100 %			
mental retardation			Global delay: 65 %	Global delay: 20 %		39 %		100 %	100 %			3 %	
speech delay			50 %	39 %					No speech				
motor delay			50 % (7/14)	76 % (26/34)					100 %				
learning difficulties									100 %			33 %	
behaviour			20 %	9 %				70-90 %	100 %			9 %	
feeding difficulties			90 %	84 %	Reported	43 %		78 %	>80 %				7 cases
seizures		1 case							>80 %				1 case
excessive sweating			75 %	64 %					Increased sensitivity to heat				
scoliosis			5 %	9 %		23 %		40-80 % [88]	<80 %				1 case
adipositas				Reported in later life [21]		yes		67 %	<80 %				
dysmorphic/typical facial gestalt	18 % (21/114)		Triangular face				100 %		>80 %		14.2 % (6/49)		Mild
clinodactyly/finger abnormalities	8 % (9/116)		45 %	75 %									5 cases
ear abnormalities			Low set posterior	Low set posterior	61.8 % (230/372)								
otitis media			20 %	14 %		17.6 % (9/51)							
hepatoblastoma					Reported		Reported						
cardiac anomalities	9 % (10/114)			9 %	5-10 % [39]								

^a^See http://www.fish.uniklinikum-jena.de/UPD.html (15.06.2015)

Currently, nine IDs have been described, but there are certainly more: In addition to the generally accepted paediatric IDs and the specific precocious puberty entity, there are three further molecular disturbances in discussion to represent separate IDs (upd(6)mat, upd(16)mat, upd(20)mat).

### Transient neonatal diabetes mellitus type 1

Transient neonatal diabetes mellitus type type 1 (TNDM1) is characterised by intrauterine growth retardation (IUGR) and hyperglycaemia in infancy. The diabetes mellitus typically develops in the first weeks of life and resolves by the age of 18 months; however, it is growing clear that individuals with TNDM are at risk of relapse, in adolescence or early adulthood, with type 2 diabetes [16, 17]. Aside from these features, TNDM1 has no major cardinal features; however, individuals may have congenital abnormalities [18]. Macroglossia (large tongue) affects just under half of infants with TNDM1, and about one in five individuals may also have a minor anomaly of the abdominal wall. Other congenital problems are rare and may be associated with MLID rather than TNDM *per se*. Approximately 10 % of individuals with TNDM1 do not present with hyperglycaemia at birth [19].

TNDM is associated with an overexpression of *PLAGL1*/ZAC in 6q24 (Fig. 1), a maternally imprinted gene. It encodes a zinc finger protein which binds DNA and hence influences the expression of other genes (for review, [20].

### Silver-Russell syndrome

SRS is a clinically and molecularly heterogeneous growth retardation syndrome. Apart from pre- and postnatal growth failure, the major features include a relative macrocephaly at birth, a typical facial gestalt (protruding forehead, triangular face), body asymmetry, and feeding difficulties in infancy. Furthermore, first follow-up data indicate a risk for adult-onset diseases [21]. The clinical presentation is variable and at least in part influenced by the mosaic distribution of molecular changes [22], but several scoring systems have been suggested [23]. Approximately 10 % of SRS patients have maternal UPD for chromosome 7 (upd(7)mat) or segmental upd(7q)mat (for review, [24, 25]) (Fig. 2). The majority of patients carry molecular changes in 11p15, the most prevalent (~40 %) being hypomethylation of *H19/IGF2*: IG DMR (Fig. 3). Additionally, numerous (submicroscopic) disturbances of chromosomes 7 and 11 as well as of other chromosomes have been described; thus screening for cryptic genomic imbalances is indicated after exclusion of upd(7)mat and 11p15 epimutations [26, 27]. The genes causing the SRS phenotype on chromosomes 7 and 11 are currently unknown, but evidences for a role of *IGF2* and *CDKN1C* in 11p15.5 and *MEST* in 7q32 have been reported [9, 28–30].

### Beckwith-Wiedemann syndrome

BWS was initially called EMG syndrome from its three main features of exomphalos, macroglossia and (neonatal) gigantism. The clinical diagnosis of BWS is often difficult due to its variable presentation and the phenotypic overlap with other overgrowth syndromes (for review, [31–33]) and isolated hemihypertrophy. In 5–7 % of children, embryonal tumours (most commonly Wilms tumour) are diagnosed.

In nearly 80 % of BWS patients chromosome 11p15.5 epimutations or mutations (Fig. 4), involving multiple loci, can be detected (including the ICR1 and *KCNQ1OT1: TSS DMR* DMRs)(for review, [34]). Most BWS cases are sporadic but familial inheritance is observed in up to 15 % of all cases. Microdeletions/duplications or point mutations at the ICRs are usually found in familial BWS with aberrant 11p15 methylation; for example, deletions and point mutations of OCT4/SOX4 binding sites in *H19/IGF2: IG DMR* are associated with *H19/IGF2: IG DMR* hypermethylation [5, 35, 36]. Conversely, *CDKN1C* mutations are frequent in familial cases with normal 11p15 methylation [37]. These BWS pedigrees resemble that of an autosomal dominant inheritance but with parent-of-origin dependent effects on penetrance. Most cases of BWS with an *KCNQ1OT1: TSS DMR* epimutation are sporadic but there is an association with assisted reproductive technologies [38]. Robust genotype/epigenotype-phenotype correlations have been established for BWS [35, 39, 40]: hemihypertrophy is strongly associated with upd(11)pat, exomphalos with *KCNQ1OT1: TSS DMR* hypomethylation and *CDKN1C* mutations, and, most importantly, the risk of Wilms tumour is significantly increased in *H19/IGF2: IG DMR* hypermethylation and upd(11)pat in comparison to the other molecular subgroups. By contrast, other embryonic tumors such as neuroblastoma and adrenal tumors are observed in patients with *KCNQ1OT1: TSS DMR* or upd(11)pat but at a much lower incidence. Hence, the determination of the molecular subtype is important for an individual prognosis and management. Nevertheless, the phenotypic transitions are fluid and testing for all molecular subtypes should be offered in patients with BWS features.

### Temple syndrome

Temple syndrome (TS14) was first described in 1991 in a patient with a maternal UPD of chromosome 14 [41], and after it turned out that it is a recognizable phenotype the name upd(14)mat syndrome was suggested. Meanwhile, other molecular changes have been reported as well [42, 43]; therefore, the name TS14 has been proposed [44] (Fig. 5). TS14 is mainly characterised by prenatal and postnatal growth retardation, muscular hypotonia, feeding difficulties in early childhood, truncal obesity and early onset of puberty. TS14 patients show clinical features overlapping with PWS and SRS; thus, screening for chromosome 14q32 should be performed in patients with PWS- and SRS-like phenotypes after exclusion of the specific (epi)mutations. For TS14 the role of an altered *RTL1* and *DLK1* expression has been suggested [42].

### Kagami-Ogata syndrome

The second recently defined ID is KOS14 which is mainly characterised by polyhydramnios, placentomegaly, excessive birth weight, a unique facial appearance with full cheeks and protruding philtrum, distinctive chest roentgenograms with coathanger rips and a bell-shaped thorax, abdominal wall defects (omphalocele, diastasis recti), variable developmental delay and/or intellectual disability, poor sucking usually requiring gastric tube feeding, hepatoblastoma and a mortality rate of 20–25 % in the neonate period [45].

Known causes of KOS are upd(14)pat (~65 %), epimutations affecting the *MEG3/DLK*: IG DMR and the *MEG3*: TSS DMR (~15 %) and microdeletions involving the *MEG3/DLK*: IG DMR and/or the *MEG3*: TSS DMR (~20 %) (Fig. 6). The detailed characterisation of KOS14 with deletions of different sizes has allowed the deciphering of the regulation mechanism in the 14q32 imprinted region [11, 46]: whereas deletion of the *MEG3/DLK*: IG DMR is associated with both the clinical KOS14 phenotype and placental abnormalities, carriers of deletions restricted to the *MEG3*: TSS DMR do not show placental abnormalities. It has been postulated that the increased expression of RTL1 is responsible for the clinical outcome, whereas a role of *DLK1* can be neglected [42].

### Angelman syndrome

A clinical diagnosis of AS demands fulfilment of four major criteria and minimum three of the six minor criteria. The major criteria are severe developmental delay, movement or balance disorder, severe limitations in speech and language and typical abnormal behavior including happy demeanor and excessive laughter. The six minor criteria are postnatal microcephaly, seizures, abnormal EEG, sleep disturbance, attraction to or fascination with water, and drooling [47]. The unique clinical features do not usually manifest within the first year of life, but developmental delay is noticed around 6 months of age. In about 10 % of the individuals with a clinical diagnosis of AS it is not possible to find the underlying genetic mechanism and other diagnoses should be considered. AS can be caused by maternally derived de novo deletion of 15q11-q13 (70–75 %), paternal uniparental disomy (upd(15)pat) of chromosome 15 (3–7 %) or an imprinting defect (2–3 %) all of which lead to lack of expression of maternally expressed 15q11-q13 genes (Fig. 7). Furthermore, mutations in *UBE3A* also cause Angelman syndrome (10–15 %). Imprinting defects can either be due to primary imprinting epimutations without DNA sequence alterations or due to deletions in the imprinting centre (IC) critical region (AS-SRO) [48, 49]. The 15q11-q13 chromosomal region contains imprinted genes, some of which are exclusively expressed from either of the parental alleles. Two exclusively maternally expressed genes, *UBE3A* and *ATP10A*, are located with this region: *UBE3A* encodes an E3 ubiquitin-protein ligase which is expressed exclusively from the maternal allele in human foetal brain and in adult frontal cortex [50, 51]. AS can be caused either by lack of *UBE3A* expression or by mutations in *UBE3A*. The role of the other imprinted gene, *ATP10A*, is however unclear. In individuals with deletions, UPD or imprinting defects, *ATP10A* expression is lacking, but in individuals with point mutations in *UBE3A* it is left unaffected.

### Prader-Willi syndrome

PWS is clinically characterised by severe hypotonia and feeding difficulties in early infancy, followed by excessive eating and development of morbid obesity in later infancy or early childhood. Cognitive impairment is seen in almost all individuals but varies in severity. A behavioral phenotype with temper tantrums, stubbornness, manipulative behavior and obsessive-compulsive disorder is constant. Hypogonadism in both males and females may cause genital hypoplasia and incomplete pubertal development; and most individuals are infertile. Short stature, and small hands and feet are common features. Characteristic facial features, strabismus and scoliosis are often present. Clinical diagnostic criteria for PWS have been developed [52, 53]; however, confirmation of the clinical diagnosis with molecular genetic testing is required.

PWS is caused by lack of expression of the paternally contributed 15q11-q13 genes. There are three mechanisms leading to PWS: deletion of the 15q11-q13 imprinted loci on the paternal allele (75–80 %), maternal UPD of chromosome 15 (upd(15)mat) (20–25 %) and imprinting defects (<1 %) (Fig. 8). The common breakpoint for the deletions are the same as for AS. Imprinting defects can either be due to primary epimutations without DNA sequence alterations or it can be due to small deletions in the imprinting centre (IC) critical region (PWS-SRO) [54]. The 15q11-q13 chromosomal region contains imprinted genes, some of which are exclusively expressed from either of the parental alleles. Paternally expressed genes are: *MKRN3*, *MAGEL2, NDN, PWRN1, C15orf2, SNURF-SNRPN* and several snoRNA genes (*SNORD64, SNORD107, SNORD108, SNORD109A, SNORD109B, SNORD115* and *SNORD116*). *SNORD115* and *SNORD116* are present in 47 and 24 gene copies, respectively, whilst the other snoRNA genes are single copy genes. Deficiency of *SNORD116* is thought to cause the key characteristics of the PWS phenotype [55, 56].

### Precocious puberty

Puberty is a complex biological process involving sexual maturation and accelerated growth. These processes normally initiate when pulsatile secretion of gonadotropin-releasing hormone (GnRH) from the hypothalamus begins. Early activation of the hypothalamic-pituitary-gonadal axis results in gonadotropin-dependent precocious puberty (also known as central precocious puberty, CPP; development of secondary characteristics before the age of 8 year in girls and 9 years in boys). With the advent of advanced sequencing methods, exome-sequencing of familial cases of CPP have identified genetic defects in transcripts with no previous link to hypothalamic-pituitary-gonadal regulation. Loss-of-function mutations in the Makorin ring finger 3 (*MKRN3*) were initially identified in CPP families [57–60]. Consistent with the genes imprinting status the phenotype was only present upon paternal transmission of the mutation. Subsequently, mutations in *MKRN3* have been shown to be the most frequent cause of familial CPP and they have also been detected in nearly 4 % in a cohort of 215 non-familial idiopathic CCP [61]. The *MKRN3* gene (also known as *ZNF127*) is an intronless transcript located on chromosome 15q11.2 in the PWS critical region, encoding for a protein with C3H zinc-finger and RING zinc-finger motifs. Unlike other imprinting disorders that can result from multiple mechanisms, it is currently unknown if CCP can arise from loss of *MKRN3* expression due to deletion, segmental maternal uniparental disomy or an imprinting defect.

### Pseudohypoparathyroidism

PHP is a group of disorders characterised by PTH resistance in the kidney, i.e. pseudohypoparathyroidism. Most cases of PHP belong to the type 1, i.e. are caused by genetic or epigenetic alterations at the imprinted *GNAS* locus. PHP1A comprises patients affected with resistance to PTH and TSH (and other GPCR proteins), and features of obesity and Albright hereditary osteodystrophy including short stature, brachydactyly, ectopic ossifications and mental retardation. PHP1A is caused by inactivating mutations in the maternal allele of the *GNAS* gene. Paternal *GNAS* mutations are associated with AHO, no hormonal resistance and no obesity, a constellation of features grouped under the term of pseudopseudohypoparathyroidism (PPHP) as well as with progressive osseous heteroplasia (POH). In contrast, the phenotype of most PHP1B patients is limited to renal PTH resistance [62] and in some cases, mild TSH resistance. Few patients with PHP1B display some features of Albright hereditary osteodystrophy [63]. Patients with PHP1B share a loss of methylation at the A/B differentially methylated region (DMR) of *GNAS*, likely leading to the downregulated expression of the *GNAS*-Gsα transcript in imprinted tissues (Fig. 9). Some patients carry additional epigenomic changes along the *GNAS* locus. About 20 % of PHP1B are inherited and due to deletions of *GNAS* imprinting control regions. The remaining 80 % are sporadic. A small subset is due to paternal UPD of chromosome 20q, yet the vast majority are still of unknown cause (for review: [64]). Whilst obesity and short stature are long known features of PHP1A, it became only recently apparent that growth and metabolism are affected in both paternal and maternal (epi)genetic alterations of the GNAS locus [65, 66].

## Do further imprinting disorders exist?

Despite the gaps in understanding their pathoaetiology and their clinical heterogeneity, the aforementioned IDs are meanwhile well established as they are associated with molecular changes in disease-specific loci. However, three further clinical entities have been suggested in which imprinted genes are known or suspected to be involved, and which might become IDs.

### Maternal uniparental disomy of chromosome 6 (upd(6)mat)

Maternal UPD of chromosome 6 (upd(6)mat) has been hypothesized to be associated with intrauterine growth retardation: among the 13 cases reported so far, 7 revealed a IUGR (http://www.fish.uniklinikum-jena.de/UPD.html). Indeed, homozygosity of a recessive allele causing IUGR has been discussed as the pathogenic mechanism as many patients share an isodisomic region in 6q16qter. However, not all upd(6)mat carriers presenting IUGR share this region, in one case homozygosity of a recessive *CUL7* has been identified causing 3 M syndrome, a growth retardation syndrome. However, it has been postulated that upd(6)mat might be regarded as a further imprinting disorder [67].

### Maternal uniparental disomy of chromosome 16 (upd(16)mat)

Maternal UPD of chromosome 16 (upd(16)mat) is the most often reported UPD other than upd(15). This is not surprising since risk of UPD is much higher in chromosomes involved in aneuploidies and trisomy 16 is the most common autosomal trisomy in human abortions. Trisomy 16 itself is usually lethal in non-mosaic state in the fetus, but in trisomy rescue is compatible with life. As a consequence of UPD formation by trisomy rescue, many of the reported upd(16)mat cases are associated with trisomy 16 mosaicism in the placenta (for review, http://www.fish.uniklinikum-jena.de/UPD.html). The upd(16)mat has been suspected to have clinical consequences. However, the heterogeneity of the birth defects observed suggested that the phenotype might rather be influenced by placenta insufficiency than by the UPD itself [68]. The possibility that upd(16)mat is associated with imprinting is difficult to assess due to the trisomy 16 mosaicism present in many cases. By an extensive clinical analysis of a series of mosaic trisomy 16 cases (n = 83) including upd(16)mat (n = 33), Yong et al. [69] concluded that upd(16)mat might be associated with more severe growth retardation in utero and an elevated risk of malformation. However, the role of imprinted genes on chromosome 16 contributing to the phenotype is unclear at the moment.

### Maternal uniparental disomy of chromosome 20 (upd(20)mat)

Maternal uniparental disomy of chromosome 20 (upd(20)mat) has been reported in 12 patients [70], 3 of whom also had mosaicism for complete or partial trisomy of chromosome 20. All patients with upd(20)mat had intrauterine and postnatal growth retardation, and prominent feeding difficulties with failure to thrive often requiring gastric tube feeding in the first few years of life. No dysmorphisms or congenital abnormalities or major developmental delay have been reported. So far, other types of molecular alterations have not yet been reported, and a candidate region on chromosome 20 has not yet been defined. It is striking that these patients have not been described to have features reminiscent of paternal *GNAS* loss of function mutations, although the loss of the paternal *GNAS* allele (on chromosome 20) is associated with pre- and postnatal growth defect and Albright hereditary osteodystrophy [8]. However, upd(20)mat probably presents a new imprinting disorder and its identification requires specialized molecular testing, which should be performed in patients with early-onset idiopathic isolated growth failure. In particular patients with a clinical diagnosis of SRS or TS14, but exclusion of their known molecular disturbances, are strong candidates for upd(20)mat as there appears to be significant phenotypic overlap.

## Multi-locus imprinting disturbances and the “imprinted gene network”

The clinical and molecular overlap between IDs suggests that there may be causal links between them, either by shared causes of dysregulation affecting multiple imprinted genes, or by perturbation of interactions between the products of imprinted genes.

A growing number of ID patients have been reported to exhibit multilocus imprinting disturbance or MLID which can vary depending on the tissues studied [22, 71] (Table 1). Whilst the mechanisms associated with MLID are currently unknown, they all present with underlying aberrations in allelic DNA methylation. Indeed, evidence is growing that genomic mutations are involved in the etiology of MLID; known trans-acting factors include mutations in the *ZFP57*, the *NLRP2* or the *NLRP7* genes [72–75].

Another hypothesis which explains the clinical and molecular overlap between the different IDs is the “imprinted gene network” (IGN) [76]. The existence of the IGN has been based on the observation of co-expression of imprinted transcript, as recently reported for the imprinted transcription factor *PLAGL1*, the gene responsible for TNDM [77]. Changes in imprinted gene abundance occur due to increased transcription from the active allele in a DNA methylation independent fashion [78]. Recently, additional gene networks have been described including the role of unoccupied insulin (IR) and insulin-like growth factor 1 receptor (IGF1R) signalling in the coordinated regulation of multiple imprinted genes associated with growth and development in mouse [79]. Interestingly this regulation is independent of *PLAGL1*, despite this gene being downregulated by more than 80 % in the IR and IGF1R double knockout cells.

Finally the paternally expressed non-coding RNA *IPW* located in the commonly deleted chromosome 15 region in PWS regulates the levels of maternally expressed transcripts within the imprinted cluster on chromosome 14 [80]. The transcriptional repression of the *DLK1-DIO3* locus by *IPW* is due to altered repressive histone modifications at the IG-DMR, which is independent of DNA methylation, via targeted recruitment of the histone methyltransferase G9a. These observations support the reports of affected individuals with TS14 who display PWS-like phenotypes [81], enforcing the view that phenotypes associated with some IDs may be caused by aberrant expression of imprinted genes within other imprinted loci.

## Conclusion

Recent rapid advances in the molecular and clinical pathogenesis of IDs have vividly illustrated the complexity of imprinting regulation, its vulnerability to genetic and epigenetic disturbance and its power as a paradigm of the interplay between genetics, epigenetics and phenotype. Identification of new mutational and epimutational pathways offers the potential for more precise molecular diagnosis and the development of new therapeutic regimes as the basis for a more directed and personalised medicine in IDs. At the same time, study of IDs may have impact beyond the borders of rare disorders, since they offer clear and tractable paradigms of the interplay between genetic, epigenetic and environmental variation upon human disorders spanning disturbances of growth and metabolism, diabetes and cancer.
