# Erratum to: Imprinting disorders: a group of congenital disorders with overlapping patterns of molecular changes affecting imprinted loci

**DOI:** 10.1186/s13148-016-0194-5

**Published:** 2016-03-07

**Authors:** Thomas Eggermann, Guiomar Perez de Nanclares, Eamonn R. Maher, I. Karen Temple, Zeynep Tümer, David Monk, Deborah J. G. Mackay, Karen Grønskov, Andrea Riccio, Agnès Linglart, Irène Netchine

**Affiliations:** Department of Human Genetics, RWTH Aachen, Pauwelsstr. 30, Aachen, Germany; Molecular (Epi)Genetics Laboratory, BioAraba National Health Institute, Hospital Universitario Araba, Vitoria-Gasteiz, Spain; Department of Medical Genetics, University of Cambridge and NIHR Cambridge Biomedical Research Centre, Cambridge, UK; Human Genetics and Genomic Medicine, Faculty of Medicine University of Southampton, Southampton, UK; WessexClinical Genetics Service, Princess Anne Hospital, Coxford Road, Southampton, UK; Clinical Genetic Clinic, Kennedy Center, Rigshospitalet, Copenhagen University Hospital, Glostrup, Denmark; Imprinting and Cancer Group, Cancer Epigenetic and Biology Program (PEBC), Institut d’Investigació Biomedica de Bellvitge (IDIBELL), Hospital Duran i Reynals, Barcelona, Spain; DiSTABiF, Seconda Università degli Studi di Napoli, Caserta, Italy; Institute of Genetics and Biophysics—ABT, CNR, Napoli, Italy; Endocrinology and diabetology for children and reference center for rare disorders of calcium and phosphorus metabolism, Bicêtre Paris Sud, APHP, Le Kremlin-Bicêtre, France; INSERM U986, INSERM, Le Kremlin-Bicêtre, France; INSERM, UMR_S 938, CDR Saint-Antoine, Paris, F-75012 France; Sorbonne Universites, UPMC Univ Paris 06, UMR_S 938, CDR Saint-Antoine, Paris, France; 3APHP, Pediatric Endocrinology, Armand Trousseau Hospital, Paris, France

## Erratum

Unfortunately, after publication of the original version of this article [1], it was noticed that there were some errors in Fig. 3 and Fig. 4:

- In Fig. 3, the methylation of H19/IGF2:IG-DMR hypomethylation is not correctly illustrated: the lolly pops should be empty (=unmethylated).
- In Fig. 4, the methylation of both H19/IGF2:IG-DMR hypermethylation and KCNQ1OT1:TSS-DMR hypomethylation are not correctly illustrated: in case of the H19/IGF2:IG-DMR hypermethylation the lolly pops should be filled (=methylated), and for the KCNQ1OT1:TSS-DMR, they should be empty (=unmethylated).

The corrected Fig. 3 and Fig. 4 have been included in this erratum.

**Fig. 3:**
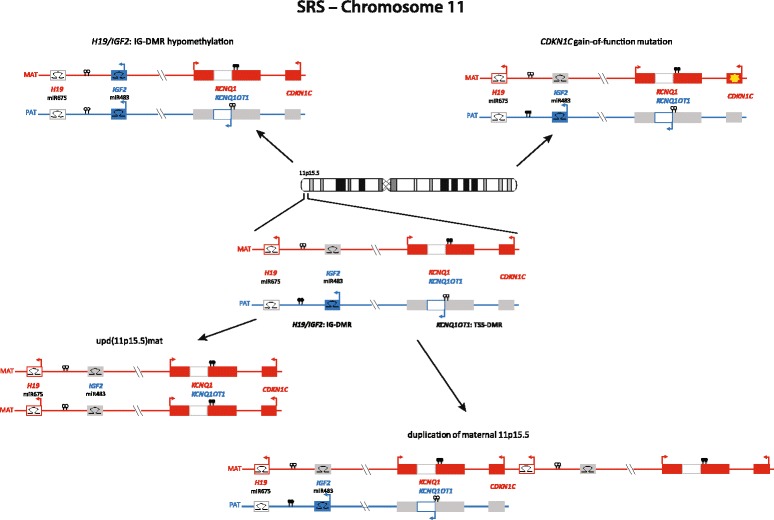
The 11p15.5 cluster can be divided in two functional domains whose imprinting is dependent on distinct imprinting control regions (*H19/IGF2: IG DMR and KCNQ1OT1: TSS DMR*). Mainly hypomethylation of the *KCNQ1OT1*: TSS DMR is responsible for SRS

**Fig. 4:**
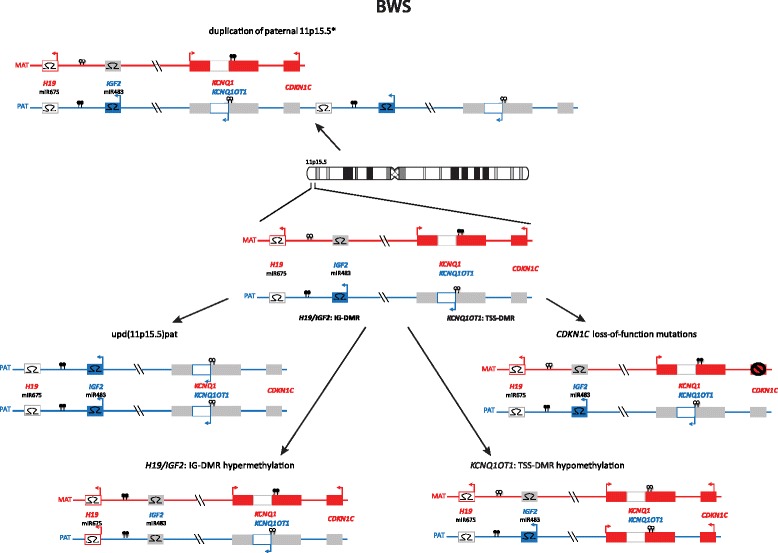
Epimutations and mutations in 11p15.5 are also responsible for BWS
